# Plasma microRNA profiles: identification of *miR-23a* as a novel biomarker for chemoresistance in esophageal squamous cell carcinoma

**DOI:** 10.18632/oncotarget.11500

**Published:** 2016-08-22

**Authors:** Shuhei Komatsu, Daisuke Ichikawa, Tsutomu Kawaguchi, Hiroki Takeshita, Mahito Miyamae, Takuma Ohashi, Wataru Okajima, Taisuke Imamura, Jun Kiuchi, Tomohiro Arita, Hirotaka Konishi, Atsushi Shiozaki, Hitoshi Fujiwara, Kazuma Okamoto, Eigo Otsuji

**Affiliations:** ^1^ Division of Digestive Surgery, Department of Surgery, Kyoto Prefectural University of Medicine, Kawaramachihirokoji, Kamigyo-ku, Kyoto, 602-8566, Japan

**Keywords:** plasma, microRNA, biomarker, prognosis, chemoresistance

## Abstract

**BACKGROUND:**

This study aims to explore novel microRNAs in plasma for predicting chemoresistance in preoperative chemotherapy of patients with esophageal squamous cell carcinoma (ESCC) using a microRNA array-based approach.

**RESULTS:**

(1) Four candidate microRNAs (*miR-223, 103a, 23b* and *23a*), which were highly expressed in the pretreatment plasma of patients with a low histopathologic response, were selected. (2) In a large-scale validation analysis by quantitative RT–PCR, plasma levels of *miR-223, miR-23b* and *miR-23a* were significantly higher in patients with a low histopathologic response than in those with a high histopathologic response (*p* = 0.0345, *p* = 0.0125 and *p* = 0.0114). (3) Of all candidate microRNAs, *miR-23a* expression of pretreatment ESCC tumor tissues was significantly higher in ESCC patients with a low histopathologic response than in those with a high histopathologic response (*p* = 0.0278). (4) After overexpressing each candidate in ESCC cells, *miR-23a* induced significant chemoresistance to both 5-fluorouracil and cisplatin, and *miR-223* to cisplatin *in vitro*. (5) A high level of plasma *miR-23a*, which tended to correlate with lymphatic invasion (*p* = 0.0808) and deep depth of invasion (*p* = 0.0658), was an independent risk factor for chemoresistance in ESCC (*p* = 0.0222; odds ratio: 12.4; range 1.46–105).

**MATERIALS AND METHODS:**

We used the Toray^®^ 3D-Gene microRNA array-based approach to compare plasma microRNA levels between patients with a high or a low histopathologic response to chemotherapy. All patients underwent a preoperative chemotherapy regimen with cisplatin plus 5-fluorouracil.

**CONCLUSIONS:**

Plasma *miR-23a* might be a useful biomarker for predicting chemoresistance in ESCC patients.

## INTRODUCTION

Esophageal carcinoma is the fifth most common worldwide cause of cancer-related death in men and is the eighth most common in women. The carcinoma constitutes a global health problem, with between 400 000 and 500 000 new cases diagnosed annually [1]. There are two histological types of esophageal carcinoma. Esophageal squamous cell carcinoma (ESCC) is the predominant histological type in Asian countries and accounts for approximately 90% of esophageal carcinomas [2]. It is one of the most aggressive carcinomas of the gastrointestinal tract. Although perioperative chemo and/or radiotherapy regimens, surgical techniques and perioperative management have progressed greatly, even now, ESCC continues to present patients with an extremely poor prognosis.

To improve the prognosis of patients with ESCC, combination therapies of preoperative chemotherapy or chemoradiotherapy followed by surgery have been developed and are widely practiced worldwide [3, 4]. In particular, chemotherapy is one of the important components in the treatment for ESCC. However, intrinsic and acquired drug resistance remains a major clinical obstacle to successful treatment [5]. Some patients receive the benefit of shrinkage in tumor mass and repression. However, the resistance of cancer cells to chemotherapeutic agents may result in progression of the disease and the subsequent metastasis of cancer cells [6, 7]. Nevertheless, there is currently no validated predictive biomarker for chemosensitivity or chemoresistance that is available in a clinical setting, and only a few mechanisms involved in cancer cell chemoresistance have been clarified in ESCC. Therefore, a better understanding of anticancer drug resistance mechanisms and the detection of clinically relevant biomarkers for predicting chemoresistance are needed to improve the survival of patients with this lethal disease.

MicroRNAs (miRNAs), which are small non-coding RNAs, regulate the translation of specific protein-coding genes. Since their discovery in 1993 [8], altered miRNA expression has been demonstrated to be associated with several diseases. In particular, miRNAs have been intensively studied in cancer research, and tumor miRNAs are involved in tumorigenesis and the development of various cancers [9–11]. Recently, several studies have identified that miRNAs are detectable in plasma/serum [11–14]. Tissue-derived miRNAs are resistant to endogenous ribonuclease activity because miRNAs may bind to proteins, such as the Argonaute 2 protein and high-density lipoprotein [15, 16], or may be packaged by secretory particles including apoptotic bodies and exosomes in plasma/serum [17–20]. Therefore, miRNAs can be present in a remarkably stable form [17, 21], and the expression levels of serum miRNAs are reproducible and consistent among individuals [14, 17]. Moreover, secretory vesicles, which include specific miRNAs, can function as intercellular transmitters. Specifically, secreted miRNAs from donor cells can be transferred to and function in recipient cells [22–24].

Several recent studies have identified that specific miRNAs in tumor tissues are involved in regulating anticancer drug resistance [25–27]. Also, in ESCC, several tumor miRNAs have been found to be related to drug resistance mechanisms and to predict chemosensitivity or chemoresistance [28–36]. Moreover, two miRNA candidates in serum for predicting chemoresistance have been identified in ESCC [37, 38]. However, these miRNAs were not always all candidates in blood miRNAs, and until now, there has been no comprehensive screening report on miRNA in plasma for predicting chemoresistance in ESCC. Therefore, we wished to find novel plasma miRNA biomarkers using a genome-wide miRNA array-based approach.

In this study, we selected four candidate miRNAs (*miR-223*, *23b*, *103a* and *23a*), which were shown by a plasma miRNA array-based approach to be more highly expressed in the pretreatment plasma of patients with a low histopathologic response than in that of patients with a high histopathologic response to preoperative chemotherapy. Finally, we validated that plasma *miR-23a* might be a useful biomarker for predicting chemoresistance in ESCC. Our results provided evidence that plasma *miR-23a* levels contribute to clinical decision-making in ESCC treatments to a clinically satisfactory degree.

## RESULTS

### Study design to detect novel plasma miRNA biomarkers of chemoresistance in ESCC

**Figure 1:**
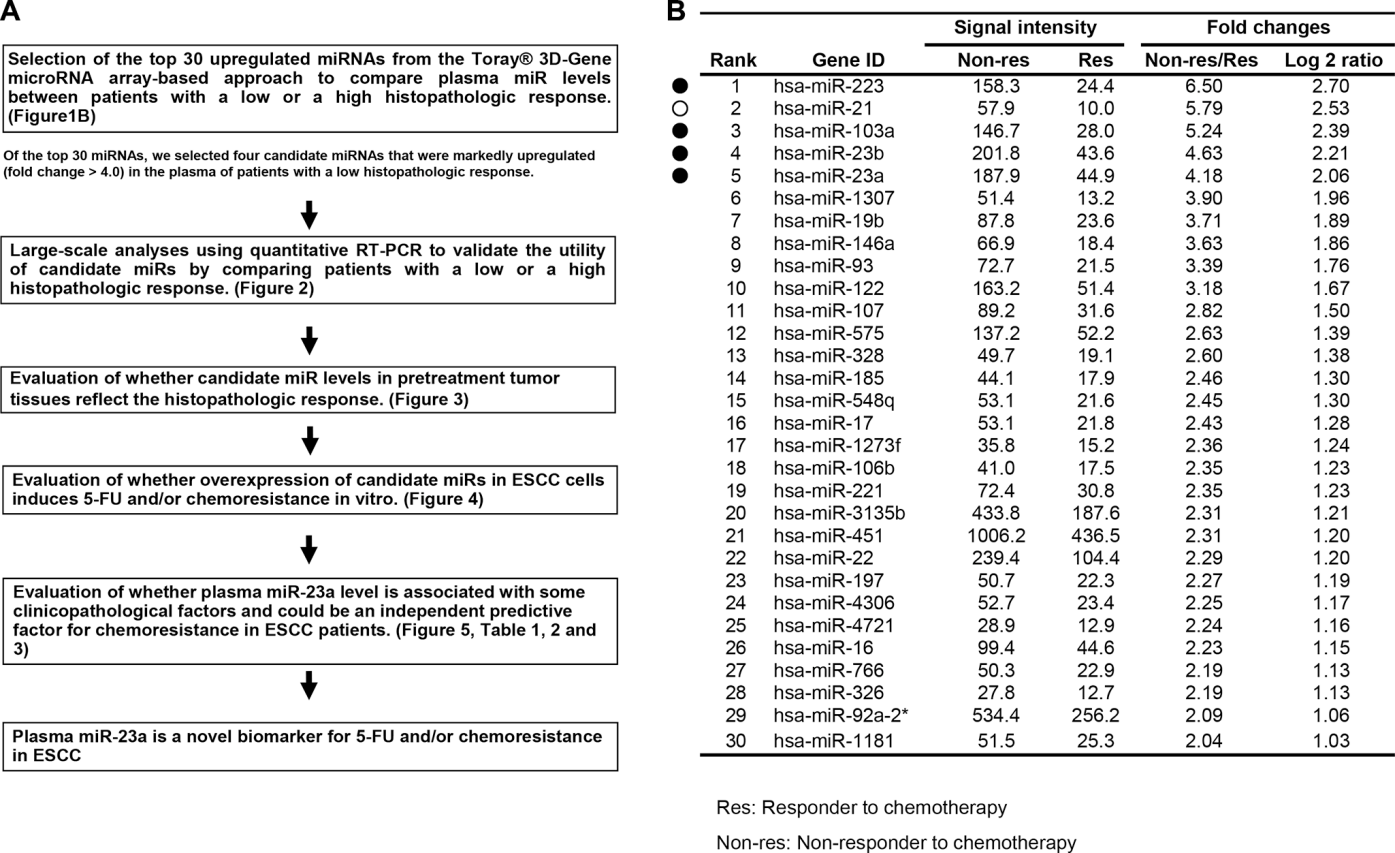
(**A**) Study design to detect novel plasma miRNA biomarkers of chemoresistance in ESCC. (**B**) Selection of plasma miRNA candidates from the comprehensive miRNA array-based approach. Using the Toray^®^ 3D-Gene miRNA array-based approach to compare plasma miRNA levels between patients with a high or a low histopathologic response, the top 30 upregulated miRNAs (fold change > 2.0) were selected. Of these, four miRNAs (closed circles) excluding *miR-21* (open circle) showing remarkable upregulation (fold change > 4.0) were selected for further analysis.

**Figure 2:**
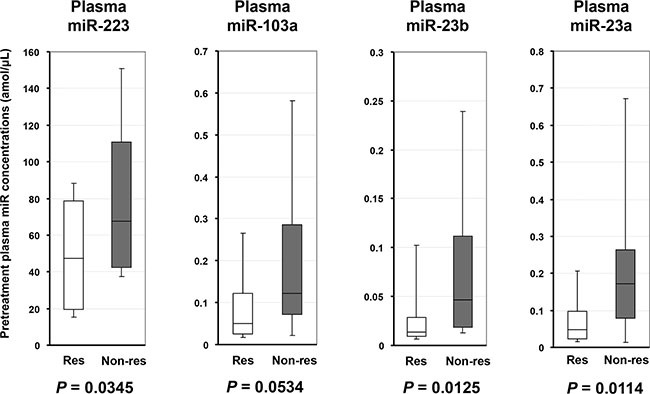
Large-scale analyses using qRT-PCR to validate the utility of candidate miRNAs by comparing patients with a low or a high histopathologic response The plasma levels of *miR-223* (*p* = 0.0345), *miR-23b* (*p* = 0.0125) and *miR-23a* (*p* = 0.0114) were significantly higher, and *miR-103a* (*p* = 0.0534) tended to be higher in the plasma of ESCC patients with a low histopathologic response than in those with a high response to chemotherapy. Res: Responder to chemotherapy; Non-res: Non-responder to chemotherapy.

**Figure 3:**
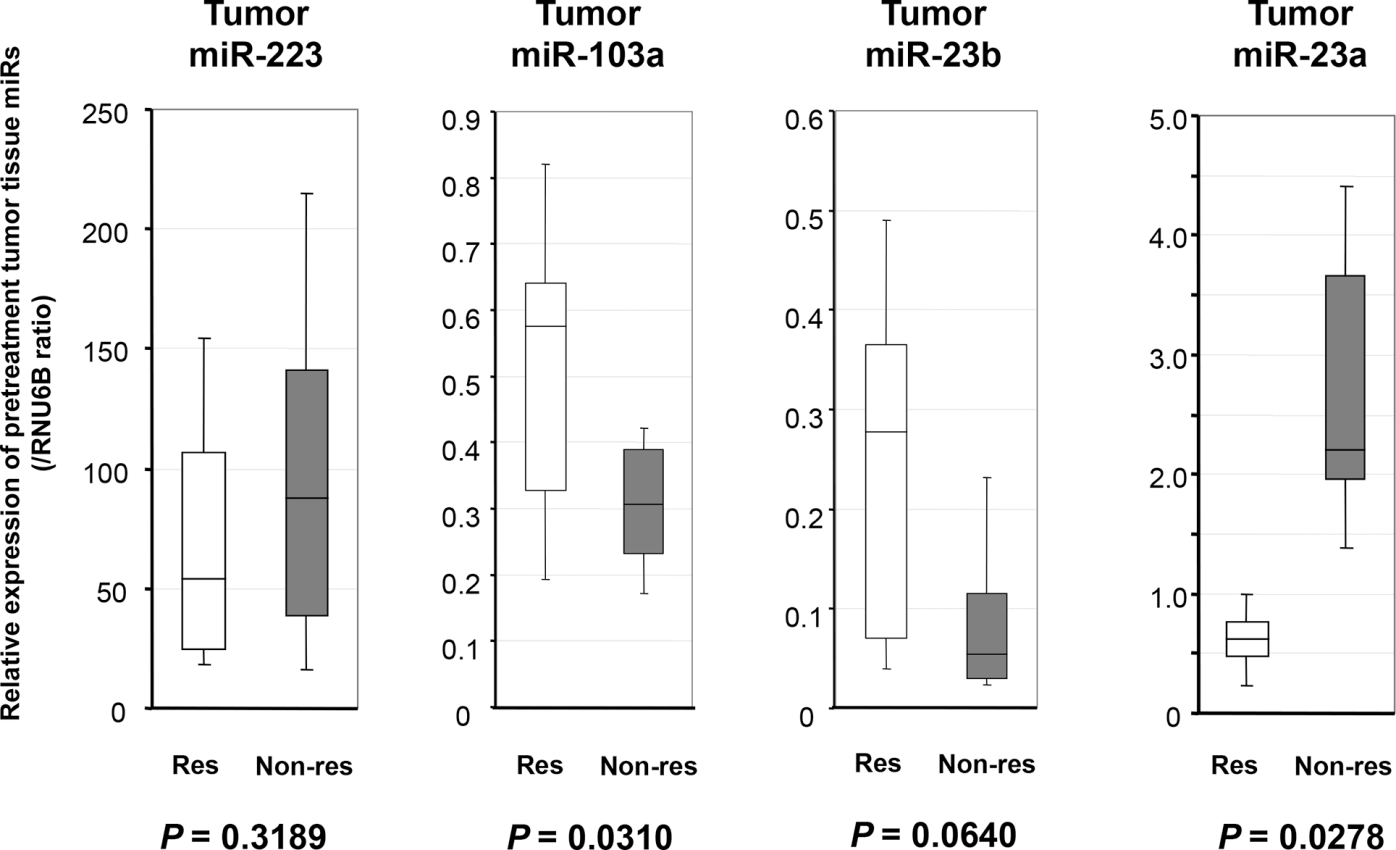
Evaluation of whether candidate miRNAs levels in pretreatment ESCC tumor tissues reflect the histopathologic response The expression of *miR-23a* was significantly higher in the pretreatment biopsy ESCC tumor tissues of patients with a low histopathologic response than in those with a high histopathologic response (*p* = 0.0278). The expressions of *miR-103a* and *miR-23b* were lower in the pretreatment biopsy ESCC tumor tissues of patients with a low histopathologic response than in those with a high histopathologic response (*p* = 0.0310, *p* = 0.0640, respectively). The results are shown after normalization to the expression of RNU6B. Res: Responder to chemotherapy; Non-res: Non-responder to chemotherapy.

**Figure 4:**
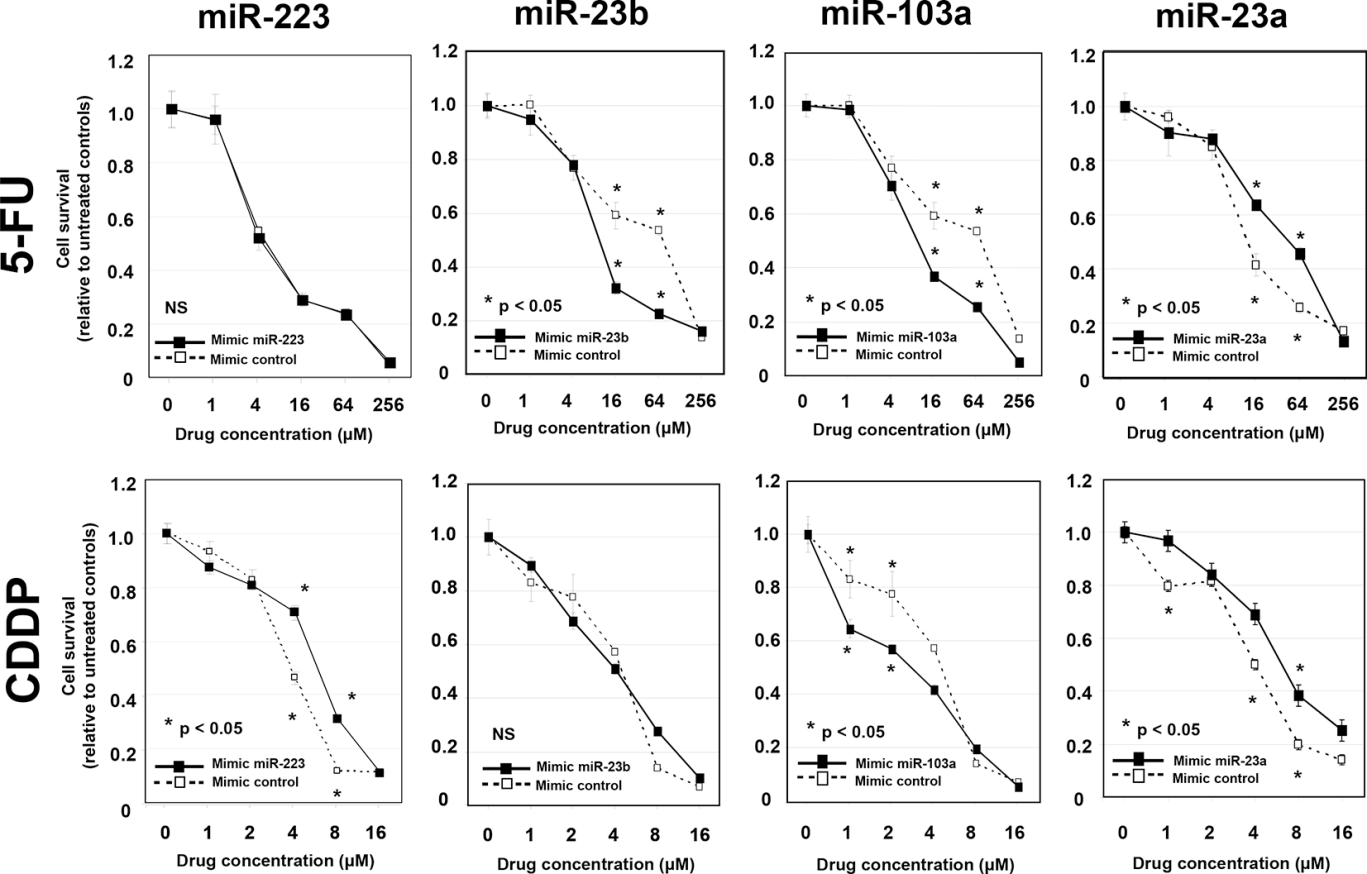
Evaluation of whether overexpression of candidate miRNAs in ESCC cells induces 5-FU and/or cisplatin chemoresistance *in vitro* After confirming the overexpression of each miRNA, the transfected KYSE170 cells were then treated with increasing concentrations of 5-FU or cisplatin, and cell viability was measured using the WST-8 assay. The viability of KYSE170 cells transfected with the control mimics was markedly inhibited by 5-FU or cisplatin. Concerning 5-FU, the inhibitory effect was significantly reduced in *miR-23a*-transfected ESCC cells. With cisplatin, the inhibitory effect was significantly reduced in *miR-223-* and *miR-23a-*transfected ESCC cells.

**Figure 5:**
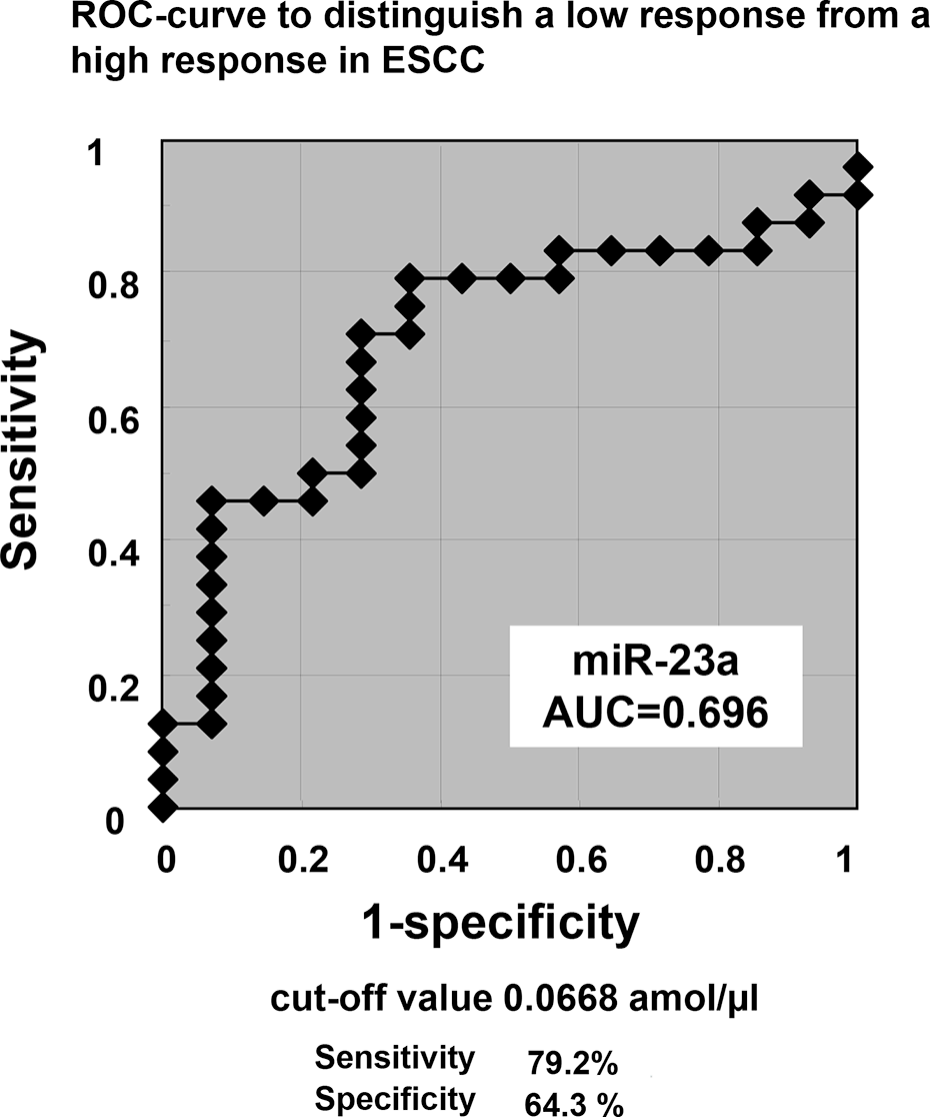
Determination of the cut-off value for the plasma *miR-23a* level that predicts chemoresistance A representation of the data using a ROC plot showed a strong separation between the low and the high pathological response groups, with an AUC of 0.696. In this model, to predict chemoresistance in clinical setting, an optimal cut-off point was indicated at 0.0668 amol/μl with a sensitivity of 79.2% and a specificity of 64.3%.

This study was divided into several parts (Figure 1A): (1) Of the top 30 upregulated miRNAs (fold change > 2.0) from the Toray^®^ 3D-Gene miRNA array-based approach to compare plasma miRNA levels between patients with a high or a low histopathologic response, four candidate miRNAs showing remarkable upregulation (fold change > 4.0) were selected (Figure 1B); (2) Large-scale analyses using quantitative RT-PCR to validate the utility of candidate miRNAs by comparing patients with a low or a high histopathologic response (Figure 2); (3) Evaluation of whether candidate miRNAs levels in pretreatment ESCC tumor tissues reflect the histopathologic response (Figure 3); (4) Evaluation of whether overexpression of candidate miRNAs in ESCC cells induces 5-FU and/or chemoresistance *in vitro* (Figure 4); (5) Evaluation of whether plasma miR-23a level is associated with some clinicopathological factors (Table 1), and whether it could be an independent predictive factor for chemoresistance in ESCC patients (Table 2).

**Table 1:** Association between plasma miR-23a levels and clinicopathological factors

Variables	Patients (*n* = 37)	Plasma miR-23a	*p*-value^b^
		(mean, amol/μl)^b^	
Age			
< 65	19 (51%)	0.1946	0.3489
≤ 65	18 (49%)	0.2024	
Sex			
Male	30 (81%)	0.1834	0.1910
Female	7 (19%)	0.2625	
Venous invasion^a^			
v0	24 (65%)	0.1266	0.1072
v1–3	13 (35%)	0.3308	
Lymphatic invasion^a^			
ly0	18 (49%)	0.1375	0.0808
ly1–3	19 (51%)	0.2561	
*p*T-stage^a^			
T0-1	10 (27%)	0.1196	0.0658
T2	8 (22%)	0.1437	
T3	17 (46%)	0.1831	
T4	2 (5%)	0.9403	
*p*N-stage^a^			
N0	12 (32%)	0.2150	0.1074
N1	14 (38%)	0.1317	
N2	5 (14%)	0.1189	
N3	6 (16%)	0.3870	
*p*Stage^a^			
I	4 (11%)	0.1595	0.1830
II	14 (38%)	0.1177	
III	16 (43%)	0.2495	
IV	3 (8%)	0.3541	

a TNM classification.

b The Mann-Whitney *U*-test and Kruskal Wallis *H*-test were performed to compare plasma miRNA concentrations.

*p*-value was considered significant at 0.05.

**Table 2:** Correlation between the histopathological responses and clinicopathological features

	Histopathological responses			
	*n*	Low (*n* = 24)	High (*n* = 13)	^a^*P* value
Sex				
male	30	20 (83%)	10 (77%)	
female	7	4 (17%)	3 (23%)	0.6779
Age				
< 65	19	12 (50%)	7 (54%)	
≤ 65	18	12 (50%)	6 (46%)	0.9036
Lymphatic invasion				
negative	18	8 (33%)	10 (77%)	
positive	19	16 (67%)	3 (23%)	**0.0170**
Venous invasion				
negative	24	14 (58%)	10 (77%)	
positive	13	10 (42%)	3 (23%)	0.3051
Depth of invasion				
cT0–T2	9	6 (25%)	3 (23%)	
cT3–T4	28	18 (75%)	10 (77%)	1.0000
Depth of invasion				
pT0–T2	18	10 (42%)	8 (62%)	
pT3–T4	19	14 (58%)	5 (38%)	0.4179
Lymph node metastasis				
cN0	9	6 (25%)	3 (23%)	
cN1-3	28	18 (75%)	10 (77%)	1.0000
Lymph node metastasis				
pN0	12	6 (25%)	6 (46%)	
pN1–N3	25	18 (75%)	7 (54%)	0.3449
pStage				
I/II	18	9 (38%)	9 (69%)	
III/III	19	15 (63%)	4 (31%)	0.1338
Pretreatment plasma miR-23a				
low	14	5 (21%)	9 (69%)	
high	23	19 (79%)	4 (31%)	**0.0109**

a *P* values are from the X^2^ or Fisher's exact test and were significant at 0.05.

NOTE: Significant values are in bold.

### Selection of plasma miRNA candidates from the comprehensive miRNA array-based approach

Of the 1719 candidates analyzed using the miRNA array-based approach, the expression levels of 266 plasma miRNAs were more highly unregulated in ESCC patients with a low histopathologic response than in those with a high histopathologic response (Supplementary Table S1). The expression levels of top 30 plasma miRNAs were more than two-fold higher in ESCC patients with a low histopathologic response than in those with a high histopathologic response (Figure 1B). In order to find more sensitive biomarkers, of these 30 miRNAs we focused on the top five miRNAs, *miR-223*, *21*, *103a*, *23b* and *23a*, with the highest expression levels of more than four-fold (i.e. with more than a two-fold log2 ratio) in the plasma of ESCC patients with a low histopathologic response (Figure 1B). We excluded *miR-21* from further analysis, because it had already been reported as a promising plasma biomarker candidate for chemoresistance in various cancers, and we have also reported [39] its usefulness for ESCC, including its diagnostic and prognostic value [40, 41]. Therefore, in this study, we selected four candidate miRNAs (*miR-223*, *103a*, *23b* and *23a*) for further analysis.

### Large-scale analyses using quantitative RT-PCR to validate the utility of candidate miRNAs by comparing patients with a low or a high histopathologic response

Next, we investigated the plasma levels of the four selected miRNAs in 37 ESCC patients by qRT-PCR. As indicated in the results from the miRNA array-based approach, the plasma levels of *miR-223* (*p* = 0.0345), *miR-23b* (*p* = 0.0125) and *miR-23a* (*p* = 0.0114) were validated to be significantly higher, and *miR-103a* (*p* = 0.0534) tended to be higher in the plasma of ESCC patients with a low histopathologic response than in those with a high response to chemotherapy (high response: Grades 3, 2, 1b; low response: Grades 1a, 0; Figure 2).

### Evaluation of whether candidate miRNAs levels in pretreatment ESCC tumor tissues reflect the histopathologic response

To confirm the high expression of selected candidate miRNAs in the pretreatment biopsy ESCC tumor tissues of patients with a low histopathologic response, we investigated the expression of each candidate miRNA according to the histopathologic response grade (high response: Grades 3, 2, 1b; low response: Grades 1a, 0) to chemotherapy using qRT-PCR. The results are shown after normalization to the expression of RNU6B. As a result, the expression of miR-23a was significantly higher in the pretreatment biopsy ESCC tumor tissues of patients with a low histopathologic response than in those with a high histopathologic response (*p* = 0.0278), and this result was coincident with the expression levels in plasma (Figure 3). Unexpectedly, the expressions of *miR-103a* and *miR-23b*, which were higher in the plasma of patients with a low histopathologic response, were inversely lower in the pretreatment biopsy ESCC tumor tissues of patients with a low histopathologic response than in those with a high histopathologic response (*p* = 0.0310, *p* = 0.0640, respectively). The detailed mechanisms behind these discrepancies were unclear.

### Evaluation of whether overexpression of candidate miRNAs in ESCC cells induces 5-FU and/or cisplatin chemoresistance *in vitro*

To determine the effects of overexpression of each candidate miRNAs on chemoresistance to 5-FU or cisplatin, the mimics of each miRNA were transfected into KYSE 170 cells. After confirming the overexpression of each miRNA, the transfected KYSE170 cells were then treated with increasing concentrations of 5-FU or cisplatin, and cell viability was measured using the WST-8 assay. The viability of KYSE170 cells transfected with the control mimics was markedly inhibited by 5-FU or cisplatin. Concerning 5-FU, the inhibitory effect was significantly reduced in miR-23a-transfected ESCC cells; whereas for cisplatin, the inhibitory effect was significantly reduced in *miR-223-* and *miR-23a-*transfected ESCC cells. Moreover, overexpression of *miR-23a* also induced significant chemoresistance to both 5-fluorouracil and cisplatin in TE5 and TE9 cells (Supplementary Figure S1). Unexpectedly, the inhibitory effect of 5-FU was significantly increased in miR-*23b-* and *miR103a -*transfected ESCC cells, and the inhibitory effect of cisplatin was significantly increased in *miR-103a*-transfected ESCC cells. The plasma levels of *miR-23b* and *miR-103a* were higher in patients with a low histopathological response. However, overexpression of these miRNAs in ESCC cells increased the chemosensitivity. The detailed mechanisms behind these discrepancies were unclear (Figure 4).

From the above analyses, we concluded that *miR-23a* was the most promising candidate in this study, because the *miR-23a* levels in both plasma and tumor tissue reflected the response to chemotherapy *in vivo*, and *miR-23a* had a role in chemoresistance to 5-FU and cisplatin *in vitro*.

### Correlation between the plasma level of miR-23a and clinicopathological factors in ESCC patients

We analyzed whether the plasma level of *miR-23a* was correlated with some clinicopathological factors in ESCC patients. As a result, plasma *miR-23a* level tended to be high in the presence of venous invasion (*p* = 0.1072), lymphatic invasion (*p* = 0.0808), advanced pT-stage (*p* = 0.0658) and advanced pN-stage (*p* = 0.1074; Table 1). These results suggest that plasma *miR-23a* level may also reflect the tumor progression of ESCC.

### Determination of the cut-off value for the plasma miR-23a level that predicts chemoresistance, and its use as an independent predictive biomarker

A representation of the data using an ROC plot showed a strong separation between the low and the high pathological response groups, with an AUC of 0.696 (Figure 5). In this model, for predicting chemoresistance in a clinical setting, an optimal cut-off point was indicated at 0.0668 amol/μl with a sensitivity of 79.2% and a specificity of 64.3%. We also examined the relationship between the histopathologic response to chemotherapy and the clinicopathological factors in ESCC patients. Factors such as lymphatic invasion (*p* = 0.0170) and pretreatment plasma *miR-23a* level (*p* = 0.0109) were correlated to the histopathologic response (Table 2). Multivariate logistic regression analysis revealed that a high pretreatment plasma concentration of *miR-23a* was an independent risk factor for chemoresistance (*p* = 0.0213; odds ratio: 12.4; range: 1.45–105.8; Table 3).

**Table 3:** Multivariate logistic regression analysis to detect independent risk factors for chemoresistance

Variables		Multivariate analysis^a^		
		OR^b^	95% CI^c^	*p* value^d^
Sex	male *vs.* female	4.132	0.264–62.50	0.3116
Age (years old)	65 ≤ *vs*.< 65	0.575	0.066–4.986	0.6157
Lymphatic invasion	positive vs.negative	12.19	1.212–125.0	**0.0337**
Venous invasion	positive vs.negative	3.003	0.290–31.25	0.3559
Depth of invasion	pT3-T4 vs.pT0-T2	4.565	0.303–68.69	0.2723
Lymph node metastasis	pN1-N3 vs.pN0	3.779	0.224–58.48	0.3414
Pretreatment plasma miR-23a	High vs.Low	12.41	1.455–105.8	**0.0213**

a Multivariate survival analysis was performed using logistic regression analysis.

b OR: Odds ratio.

c CI: confidence interval.

d *p*-value < 0.05 was significant.

NOTE: Significant values are in bold.

### Pretreatment plasma miR-23a levels in patients with Stage IV ESCC

Finally, we investigated pretreatment plasma *miR-23a* levels in 16 consecutive patients with Stage IV ESCC. All patients completely underwent 5-FU +CDDP chemotherapy as a first-line regimen until the progressive disease. Patients with Stage IV ESCC were divided into two groups such as the short-term and long-term survivors by median survival time (MST: 312 day) of all stage IV patients (A). The pretreatment plasma *miR-23a* level of short-term survivors was significantly higher than that of long-term survivors (*P* = 0.0278) (B), suggesting that high plasma miR-23a level might be an indicator for poor prognosis and be related to potential low response to chemotherapy in ESCC patients (Supplementary Figure S2).

## DISCUSSION

In the present study, we identified the plasma miRNA *miR-23a* as a predictive biomarker for chemoresistance in ESCC. This was achieved through genome-wide miRNA profiling of the plasma of ESCC patients using high-resolution miRNA arrays. The *miR-23a* expressions of pretreatment plasma and ESCC tissues were significantly higher in ESCC patients with a low histopathologic response than in those with a high histopathologic response. *In vitro*, overexpression of *miR-23a* was proved to induce significant chemoresistance to both 5-FU and cisplatin. Furthermore, a high level of plasma *miR-23a*, which tended to correlate with tumor progression, was an independent risk factor for chemoresistance in ESCC patients (odds ratio: 12.4). These findings strongly suggest that *miR-23a* might have a pivotal role in tumor development and chemoresistance in ESCC. Also, plasma *miR-23a* level may contribute to the decision-making process for chemotherapy in ESCC patients, to a clinically satisfactory degree of sensitivity and specificity.

Several studies have identified that the aberrant expression of *miR-23a* occurs in various types of cancer. *miR-23a*, which is located in *the miR-23a~27a~24-2* cluster (19p13; [42–44]), has mainly an oncogenic function and is frequently overexpressed in various malignancies [42] such as acute lymphoblastic leukemia (ALL; [45]), acute myeloid leukemia (AML; [45]), breast cancer [44], gastric cancer [46, 47], hepatocellular carcinoma (HCC; [43]), lung cancer [48], pancreatic cancer [49, 50], colorectal cancer [51, 52], ovarian cancer [53], laryngeal cancer, glioma [54, 55], tongue squamous cell carcinoma (TSCC; [56]), oral squamous cell carcinoma (OSCC; [57]), bladder cancer, papillary thyroid carcinoma and osteosarcoma [58], through directly targeting various tumor-suppressor genes such as *PTEN* [58], *CDH1* [48], *IRF1* [59], *APAF1* [49, 55], *HOXD10* [54] and *MTSS1* [51]. In contrast, miR-23a is downregulated in prostate cancer [60]. Concerning *miR-23a* in chemoresistance, *miR-23a* has already been reported as having pivotal functions in the chemoresistance to cisplatin in TSCC [56] and ovarian cancer [53] through inhibiting TOP2B which is a target of miR-23a and functions as the target for several anticancer agents [56], and to paclitaxel in gastric cancer through the suppression of paclitaxel-induced apoptosis by targeting IRF1 at the post-transcriptional level [59]. Therefore, *miR-23a* might have various crucial roles in carcinogenesis, tumor development and chemoresistance in ESCC as well as other cancers.

Regarding the plasma *miR-23a* as a potential marker available for other clinical applications excepting chemoresistance in combination chemotherapy of 5-FU and cisplatin for ESCC, the function of *miR-23a* for chemoresistance to paclitaxel was already reported in gastric cancer [59]. Generally, 5-FU, cisplatinl and taxane are normally used as key drugs for ESCC chemotherapy. Therefore, plasma *miR-23a* level may be also useful to predict chemoresistance for taxane in ESCC. However, there is currently no clinical data associated with plasma *miR-23a* level in ESCC. Moreover, plasma *miR-23a* may be a biomarker for early detection of ESCC. Previously, we investigated microRNAs in plasma for cancer detection through comprehensive genome-wide microRNA array-based approach to compare plasma microRNA levels between ESCC patients and healthy volunteers [59, 61]. As a result, plasma *miR-23a* level of ESCC patients was significantly higher than healthy volunteers (fold change 2.17). Thus, plasma *miR-23a* might be a useful biomarker for predicting chemoresistance and cancer detection in ESCC.

Eight ESCC-associated miRNAs have been reported in ESCC tissues in response to chemotherapy: *miR-27a*, *miR-200c*, let-7, *miR-483*, *miR-214*, *miR-113a/b*, *miR-24* and *miR-634* [28–36]. In contrast, two blood-based biomarkers have been reported in serum: *miR-200c* and *miR-27* [37, 38]. Specifically, Tanaka *et al.* first investigated four serum miRNAs (*miR-21*, *miR-145*, *miR-200c* and *let-7c*) in cisplatin-based chemotherapy, which had already been determined as candidate miRNAs in cancer tissues associated with chemoresistance or prognosis [31]. As a result, serum *miR-200c* was selected as a candidate biomarker [38]. Moreover, they then performed an analysis using the Toray^®^ 3D-Gene miRNA microarray to compare the serum miRNA levels in patients with a low or a high histopathologic response. Among the 18 candidates showing a change in expression of more than 1.7-fold, the high expression of *miR-27a/b* in serum was proved to be correlated to chemoresistance and poor prognosis because *miR27a/b* could induce chemoresistance of the cancer stroma *in vitro* [37].

In this study, we investigated plasma miRNA profiles using a high-resolution miRNA array-based approach to compare plasma miRNA levels between patients with a low or a high histopathologic response. The study is justified by there being no reports on plasma miRNAs for chemoresistance in ESCC through the comprehensive array-based approach. Regarding plasma miRNAs, previous studies concerning blood miRNA profiles revealed that the majority of circulating miRNAs were co-fractionated with plasma protein complexes [16]. Moreover, compared to serum, plasma might retain higher protein levels, including those of cogulant-related proteins. Indeed, in our study, more remarkable changes were detected in plasma miRNAs between ESCC patients with a low or a high histopathologic response than were detected in serum miRNAs [37]. Specifically, in our study, 58 candidate miRNAs (fold range: 1.70–6.50) presenting more than a 1.7-fold change in chemoresistance (Supplementary Table S1) were detected in plasma miRNA profiles, in comparison with 18 candidate miRNAs (fold range: 1.70–2.41) derived from serum miRNA profiles [37]. Moreover, the detected candidate miRNAs between plasma and serum were considerably different [37]. Indeed, of the miRNAs presenting more than a 1.7-fold change in both plasma and serum miRNA microarray studies, only six miRNAs (*miR-223*, *19b*, *107*, *451*, *16* and *25*) were commonly detected. This phenomenon was also detected in studies of the diagnostic blood-based miRNA candidates in ESCC [61, 62] and pancreatic cancer [63, 64]. These findings strongly suggest that plasma miRNAs as well as serum miRNAs could be useful blood-based biomarkers, and we should consider the kinds of blood samples such as serum, plasma and all blood for better clinical application of miRNAs as a blood-based biomarker.

Other striking findings in this study that both *miR-103a* and *miR-23b* showed opposite behavior in plasma and tumor tissues among four candidate miRNAs (Figure 2 and Figure 3). Both *miR-103a* and *miR-23b* have been mainly reported as a tumor suppressive miRNA [65–67]. As shown in Table 3, ESCC patients with a low level of these tumor suppressive miRNAs in ESCC tumor tissues had a low histopathologic response. These results in tumor tissues may be presumable as a function of tumor suppressive miRNAs. However, the mechanism that patients with a low histopathologic response had a high plasma level of *miR-103a* and *miR-23b* is currently unclear. Previously, we reported that some tumor suppressive miRNAs such as *miR-451* and *miR-486*, which were down-regulated in tumor tissues, presented extremely high levels in plasma of patients with tumor than those without tumor [68]. These discrepancies are very curious and may be important mechanisms in the tumorigenesis and pathogenesis of chmoresistance. Currently, these issues are under evaluation.

In summary, this is the first report to demonstrate the clinical utility of plasma *miR-23a* in ESCC. Nevertheless, many issues must be addressed before these findings can be translated into a clinically useful, non-invasive predictive strategy for chemotherapeutic response in ESCC patients. Specifically, two independent cohorts' analyses and large-scale analysis may be needed. Therefore, we will prospectively confirm the usefulness of plasma *miR-23a* as a multi-center study using a large number of patients. Also, previous reports suggested that *miR-23a* has various crucial roles in carcinogenesis, tumor development and chemoresistance in other cancers. Therefore, the clinical utility of plasma *miR-23a* may be applicable in other cancers. Furthermore, additional sensitive candidate miRNAs should be identified as liquid-based biomarkers for predicting chemoresistance of ESCC by using strategies with different body fluids and high-throughput platforms, such as next-generation sequencing or digital PCR-based approaches. These strategies are currently under evaluation, and we will report the findings in the near future.

## MATERIALS AND METHODS

### Patients and samples

This study was approved by the Institutional Review Board of Kyoto Prefectural University of Medicine, and each subject provided written informed consent. Between March 2010 and May 2012, 37 pretreatment plasma samples and biopsy tissue specimens were collected from ESCC patients at the Kyoto Prefectural University of Medicine. All patents underwent the same and complete preoperative chemotherapy (JCOG9907 regimen) with cisplatin plus 5-fluorouracil, which was repeated twice every three weeks. A dose of 80 mg/m^2^ cisplatin was administered by an intravenous drip infusion for 2 h on day one; 5-fluorouracil was administered at a dose of 800 mg/m^2^ by continuous infusion on days one through five [4]. After preoperative chemotherapy, patients underwent curative esophagectomy. All patients were pathologically diagnosed with ESCC. Tumor stages were assessed according to the 7th Union of International Control of Cancer (UICC)/TNM classification [69]. In all cases, two pathologists agreed with pathological observations and confirmed the diagnosis. Moreover, we used pretreatment plasma samples in 16 consecutive patients with Stage IV ESCC. These ESCC patients completely underwent 5-FU +CDDP chemotherapy as a first-line regimen until the progressive disease.

Peripheral blood (7 ml) was obtained from each patient at the time before preoperative chemotherapy and from the healthy volunteers. Blood was collected from patients and healthy volunteers in sodium heparin tubes (BD Vacutainer) and was immediately subjected to the three-spin protocol (1500 r.p.m. for 30 min, 3000 r.p.m. for 5 min and 4500 r.p.m. for 5 min) to prevent contamination by cellular nucleic acids. The plasma samples were stored at − 80°C until further processing. The pretreatment ESCC biopsy specimens for each patient were fixed in formalin and embedded in paraffin for the pathological diagnosis and the expression analyses of tumor miRNAs.

### Evaluation of responses to chemotherapy

In the present study, we used the histopathologic response grade of ESCC tumors as an indication of the chemotherapeutic effect. We used this because previous studies have suggested that the histopathologic response is more strongly correlated with clinical outcomes and/or survival than is the clinical response grade by imaging modalities in ESCC [70–72]. The degree of histopathologic tumor regression in surgical specimens was classified into five categories according to the 10^th^ guidelines of the Japan Esophageal Society [73]. The percentage of viable residual tumor cells within the total cancerous tissue was assessed as follows: Grade 3, no viable residual tumor cells; Grade 2, less than 1/3 residual tumor cells; Grade 1b, 1/3 to 2/3 residual tumor cells; Grade 1a more than 2/3 residual tumor cells; Grade 0, no significant response to chemotherapy. The number of patients of each histopathologic grade was one patient with Grade 3, 6 patients with Grade 2, 6 patients with Grade 1b, 23 patients with Grade 1a and one patient with Grade 0. In the present study, we allocated 13 patients with Grades 3, 2, 1b into the high histopathologic response group and 24 patients with Grades 1a, 0 into the low histopathologic response group.

### RNA extraction

Total RNA was extracted from 400 μl of plasma using the mirVana PARIS Kit (Ambion, Austin, TX, USA) and finally eluted into 100 μl of preheated (95°C) Elution Solution according to the manufacturer's protocol. Using the formalin-fixed paraffin-embedded tissues, total RNA was extracted from four 15-μm-thick slices of tissue (total 60 μm in thickness) using the RecoverAll Total Nucleic Acid Isolation Kit (Ambion, Austin, TX, USA) and then eluted into 60 μl of Elution Solution according to the manufacturer's protocol.

### miRNA microarray analysis

Microarray analyses of each miRNA level in the pretreatment plasma samples were performed using the 3D-Gene miRNA microarray platform (Toray, Kamakura, Japan; [61, 64, 68, 74, 75]). Plasma miRNA levels were compared between three different ESCC patients with a low response grade and three different ESCC patients with a high response grade. The ESCC patients with a low histopathological response grade consisted of one patient with Grade 0 (no response to chemotherapy) and two patients with Grade 1a (more than 2/3 residual tumor cells). Imaging modalities showed no tumor regression for these patients. In addition, the ESCC patients with a high histopathologic response grade consisted of one patient with Grade 3 (no viable tumor cells) and two patients with Grade 2 (less than 1/3 residual tumor cells). Imagining modalities showed more than 50% tumor regression for these patients. Each 100 μl plasma sample from the three ESCC patients with a high response grade of Grade 3 or Grade 2 was mixed, and the total plasma sample of 300 μl was used. On the other hand, each 100 μl plasma sample from the three ESCC patients with a low response grade of Grade 0 or Grade 1a was mixed, and the total plasma sample of 300 μl was used.

RNA extraction and microarray analysis were performed according to the manufacturer's instructions described previously [68]. Briefly, the amount of total RNA in plasma was too small, and so 2 of 4 μl of extracted total RNA from 300 μl of plasma samples were used in the microarray experiments. This RNA was labeled with Hy5 using the Label IT miRNA Labeling Kit (Takara Bio, Otsu, Japan) and hybridized at 32°C for 16 h on the 3D-Gene chip. The 3D-Gene miRNA microarray (Human_miRNA_17v1.0.0, Toray Industries) can mount > 1500 miRNAs based on the Human miRNA Version17 of MirBase (http://microrna.sanger.ac.uk/). The microarray was scanned and the images obtained were numerated using 3D-GeneH scanner 3000 (Toray Industries). The expression level of each miRNA was globally normalized using the background-subtracted signal intensity of the entire set of miRNAs in each microarray. The obtained microarray images were analyzed using GenePix Pro TM (Molecular Devices, Sunnyvale, CA).

### Quantification of miRNA by qRT-PCR

The amounts of miRNAs were quantified by qRT-PCR using the human TaqMan MicroRNA Assay Kit (Applied Biosystems, Foster City, CA). The reverse transcription reaction was carried out with a TaqMan MicroRNA Reverse Transcription Kit (Applied Biosystems) in 5 μl of solution, containing 1.67 μl of extracted RNA, 0.05 μl of 100 mM dNTPs, 0.33 μl of Multiscribe Reverse Transcriptase (50 Uμl^−1^), 0.5 μl of 10× Reverse Transcription Buffer, 0.06 μl of RNase inhibitor (20 Uμl^−1^), 1 μl of gene-specific primer (hsa-miR-223, Assay ID: 002295; hsa-miR-103a, Assay ID: 000439; hsa-miR-23b, Assay ID: 000400; hsa-miR-23a, Assay ID: 000399; cel-miR-39, Assay ID: 000200; and RNU6B, Assay ID: 001093) and 1.39 μl of nuclease-free water. To synthesize cDNA, reaction mixtures were incubated at 16°C for 30 min, at 42°C for 30 min, and at 85°C for 5 min, and then were held at 4°C. Next, 0.67 μl of cDNA was amplified using 5 μl of TaqMan 2× Universal PCR Master Mix with no AmpErase UNG (Applied Biosystems), 0.5 μl of gene-specific primers/probe, and 3.83 μl of nuclease-free water in a final volume of 10 μl. Quantitative PCR was run on a StepOnePlus PCR system (Applied Biosystems), and reaction mixtures were incubated at 95°C for 10 min, followed by 40 cycles of 95°C for 15 sec and 60°C for 1 min. Cycle threshold (Ct) values were calculated with StepOne Software v2.0 (Applied Biosystems).

As previously reported [17], we used an approach for data normalization based upon spiking the sample with a synthetic RNA oligonucleotide, cel-*miR-39*, which does not exist in the human genome. *C. elegans* cel-*miR-39* was purchased as a custom-made RNA oligonucleotide (*Qiagen*, Valencia, CA). The oligo used for spiking, as a mixture of 25 fmol of oligonucleotide in 5 ul of total volume of water, was introduced after the addition of 2X Denaturing Solution (Ambion) to the plasma or serum sample to avoid degradation by endogenous plasma RNases. As a control for each RNA sample, cel-miR-39 was used for TaqMan qRT-PCR assays (Applied Biosystems) as described earlier. We normalized the data across samples using the 2^−ΔΔCt^ method relative to cel-miR-39. Whereas, expression of miRNAs from tissue samples and cultured cells was normalized using the 2^−ΔΔCt^ method relative to U6 small nuclear RNA (RNU6B). ΔCt was calculated by subtracting the Ct values of cel-*miR-39* or RNU6B from those of the miRNAs of interest. ΔΔCt was then calculated by subtracting the mean of ΔCt of plasma of healthy volunteer or normal pancreatic tissue from the ΔCt of PCa tissues. The change in gene expression was calculated with the equation 2^−ΔΔCt^ [76, 77].

### ESCC cell lines and culture

ESCC cell line, KYSE170 [78], was purchased from RIKEN Cell Bank (Tsukuba, Japan) and cultured in Roswell Park Memorial Institute (RPMI)-1640 medium (Sigma, St Louis, MO) supplemented with 10% FBS (Trace Scientific, Melbourne, Australia). This cell line was cultured in 5% carbon dioxide at 37°C in a humidified chamber.

### Oligonucleotide transfection

To overexpress each miR, the mimic (Assay ID: MC12301 (*miR-223*), MC10711 (*miR-23b*), MC10632 (*miR-103a*) and MC10644 (*miR-23a*)) or control mimic miRNA (mirVana miRNA mimic Negative Control #1) selected from the mirVana miRNA mimic panel (Ambion, Austin, TX, USA) was transfected into cells (50 μM) using Lipofectamine RNAiMAX (Invitrogen) according to the manufacturer's instructions [64]. The overexpression of each miR was confirmed by qRT-PCR using human TaqMan MicroRNA Assay Kits (Applied Biosystems, Foster City, CA, USA).

### Cell viability assays

To assess the chemoresistance of an ESCC cell line to 5-fluorouracil (5-FU) or cisplatin, KYSE170 cells that were transfected with each miRNA or its control were plated onto a 24-well plate (3 × 10^4^ cells/ml) and incubated overnight under normal culture conditions. The KYSE170 cells were then incubated with various concentrations of 5-FU (1, 4, 16, 64 or 256 μM) or cisplatin (1, 2, 4, or 16 μM). These cells were subjected to the WST-8 assay 72 h after the 5-FU treatment and 48 h after the cisplatin treatment. The number of viable cells was determined with a cell counting kit (Dojindo Molecular Technologies, Inc., Gaithersburg, MD), which counted the number of living cells using WST-8.

### Statistical analysis

For miRNA array-based analyses, the signal intensity ratio and log2 ratio of each plasma miRNA were calculated by the ratio of ESCC patients with a low histopathologic response to those with a high histopathologic response. The Mann–Whitney *U-test* for unpaired data from plasma or tissue samples was performed. The Kruskal-Wallis *H*-test was also used to compare more than two groups. The Chi-square test or Fisher's exact probability test was used to evaluate correlations between the results of plasma miRNA levels and clinicopathological factors. A *P*-value < 0.05 was considered statistically significant.

Receiver-operating characteristic (ROC) curves and the area under the ROC curve (AUC) were used to assess the feasibility of using plasma miRNA as a diagnostic tool for predicting chemoresistance in ESCC. The Youden index was used to determine the cutoff value for the plasma miRNAs levels [79]. Multivariate logistic regression analysis was performed to identify independent risk factors associated with chemoresistance. Multivariate odds ratios are presented with 95% confidence intervals.

## SUPPLEMENTARY MATERIALS FIGURES AND TABLE




