# Supplementary material for: Plasma microRNA profiles: identification of miR-23a as a novel biomarker for chemoresistance in esophageal squamous cell carcinoma
Source: Oncotarget. 2016 Aug 22;7(38):62034–48. doi: 10.18632/oncotarget.11500 (PMC5308709; doi:10.18632/oncotarget.11500)
Supplement: Supplementary file 1 [file oncotarget-07-62034-s001.pdf]

Plasma microRNA profiles: identification of *miR-23a* as a novel biomarker for chemoresistance in esophageal squamous cell carcinoma

Supplementary Materials

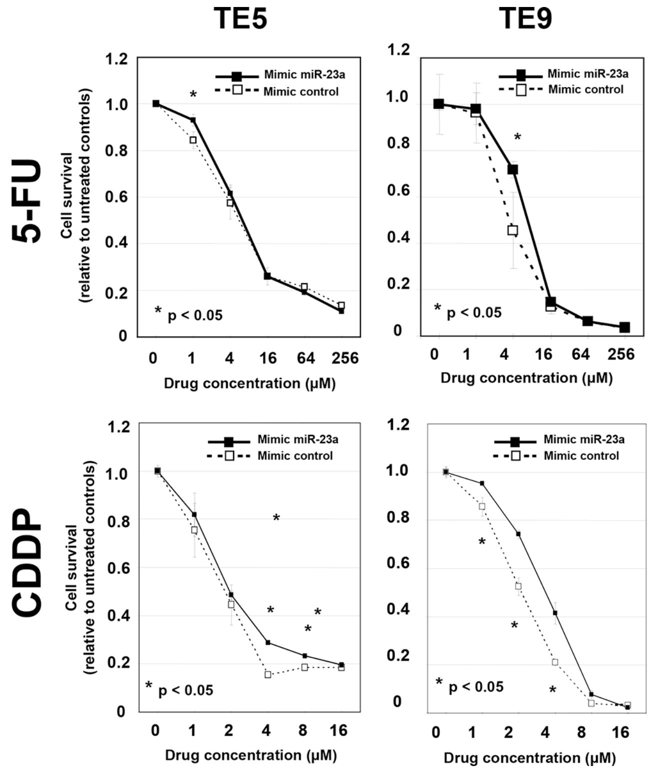

Supplementary Figure S1: Evaluation of whether overexpression of *miR-23a* in ESCC cells induces 5-FU and/or cisplatin chemoresistance in TE5 and TE9 cells. After confirming the overexpression of *miR-23a*, the transfected TE5 and TE9 cells were then treated with increasing concentrations of 5-FU or cisplatin, and cell viability was measured using the WST-8 assay. Overexpression of *miR-23a* induced significant chemoresistance to both 5-fluorouracil and cisplatin in both TE5 and TE9 cells.

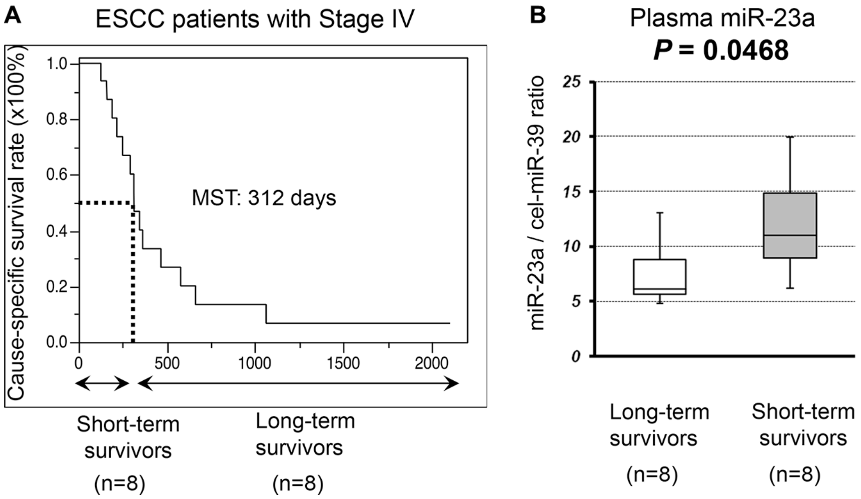

Supplementary Figure S2: Pretreatment plasma *miR-23a* levels in 16 consecutive non-curative patients with Stage IV ESCC. All patients completely underwent 5-FU +CDDP chemotherapy as a first-line regimen until the progressive disease. Patients with Stage IV ESCC were divided into two groups such as the short-term and long-term survivors by median survival time (MST: 312 day) of all stage IV patients (**A**). The pretreatment plasma *miR-23a* level of short-term survivors was significantly higher than that of long-term survivors ($P = 0.0278$) (**B**), suggesting that high plasma *miR-23a* level might be an indicator for poor prognosis and be related to potential low response to chemotherapy in ESCC patients.

Supplementary Table S1: Oncotarget plasma miR-23a in ESCC. See Supplementary_Table_S1
